# Gamma-carboxylation regulates osteocalcin function

**DOI:** 10.18632/oncotarget.5126

**Published:** 2015-08-10

**Authors:** Julie Lacombe, Mathieu Ferron

**Affiliations:** Unité de recherche en physiologie intégrative et moléculaire, Institut de Recherches Cliniques de Montréal, Montréal, Québec, Canada

**Keywords:** osteocalcin, vitamin K, GGCX, VKORC1, glucose

The only well described function of vitamin K (VK) is to serve as a co-factor for the γ-carboxylation of particular proteins to convert specific glutamic acid (Glu) residues to γ-carboxyglutamic acid (Gla) residues. This process involves two enzymes, γ-glutamyl carboxylase (γ-carboxylase or GGCX) and vitamin K epoxide reductase (VKORC1), which together constitute the vitamin K cycle [1]. Physiologically, γ-carboxylation is known to be critical for the function of many coagulation factors (prothrombin, factor IX, factor VII, etc), but is also implicated in the regulation of Matrix Gla Protein (MGP) that inhibits extra-osseous tissue mineralization and of osteocalcin that regulates glucose metabolism.

Osteocalcin is an osteoblast-derived γ-carboxylated protein present at high concentration in the bone extracellular matrix (ECM). Because Gla osteocalcin have great affinity for hydroxyapatite, the mineral component of bone ECM, it was originally believed to play a role in bone mineralization. Nevertheless, gain- and loss-of-function mouse models of osteocalcin have shown that this protein is not required for normal bone remodeling or mineralization [2]. Instead, the characterization of *Ocn*^−/−^ mice revealed that osteocalcin is acting as a bone-derived hormone regulating glucose metabolism [3-4]. Cell culture and *in vivo* studies have shown that osteocalcin improves glucose handling by promoting insulin secretion by β-cells on one hand and by favouring insulin sensitivity on the other hand [1]. Although both the Gla and the Glu forms of osteocalcin are detected in the serum, the vast majority of the *in vitro* and *in vivo* studies conducted so far suggests that the endocrine function of osteocalcin is fulfilled by its Glu form. Interestingly, additional experiments involving the characterization of mice having either increased or decreased osteoclast number suggest that bone resorption is required to decarboxylate and release osteocalcin from the bone matrix [5–6]. Accordingly, many but not all studies in human have shown that serum levels of Glu osteocalcin negatively correlate with insulin resistance, obesity, diabetes or markers of the metabolic syndrome [1]. So far the data on osteocalcin endocrine regulation were supporting a model in which osteocalcin produced by osteoblasts is stored as a γ-carboxylated and inactive protein in the bone ECM, before being activated by decarboxylation during bone resorption. This model predicts that blocking osteocalcin γ-carboxylation would prevent osteocalcin accumulation in the bone ECM and thereby improve glucose homeostasis. In a recent study published in *The Journal of Cell Biology* [7] we tested directly this assumption aiming at providing direct evidence demonstrating that γ-carboxylation acts negatively on the endocrine functions of osteocalcin.

Mice lacking *Ggcx* or *Vkorc1* globally die perinatally from severe hemorrhages, precluding the study of the role of γ-carboxylation and VK reduction on osteocalcin endocrine function in adults. Hence we generated “floxed” alleles of *Ggcx* and *Vkorc1* in mice in order to be able to inactivate each of these two genes in a tissue-specific manner. Interestingly, mice lacking *Ggcx* or *Vkorc1* in osteoblasts were viable and displayed a marked reduction in Gla osteocalcin serum levels while Glu osteocalcin levels were increased. In addition, osteocalcin failed to accumulate in the bone ECM in the *Ggcx* and *Vkorc1* osteoblast-specific knockout mice. Importantly, these mice also show a significant improvement in glucose tolerance and in insulin sensitivity and were protected from high fat diet-induced insulin resistance and obesity [7].

There is only one γ-carboxylase-encoding gene in mammals; however, all vertebrates possess a second VKORC1-like encoding gene called *VKORC1L1*. The function of this enzyme remains unclear, but it has been suggested to have VK epoxide reductase activity *in vitro*, compensating for the absence of VKORC1 in particular cell culture conditions. For this reason, we also generated a conditional allele of this gene in the mouse. Our characterization of the global and osteoblast-specific *Vkorc1l1* deficient mice suggests that VKORC1L1 is not implicated in osteocalcin carboxylation. In addition, deleting *Vkorc1l1* in osteoblast-specific *Vkorc1*-deficient mice did not further affect the circulating levels of Glu osteocalcin or osteocalcin bone content when compared to *Vkorc1*-osteoblasts knockout littermates [7]. These results suggest that VKORC1L1 cannot compensate for VKORC1 absence with respect to γ-carboxylation in osteoblasts. However, it cannot be excluded that VKORC1 and VKORC1L1 may have redundant function(s) in osteoblasts independently of γ-carboxylation.

Altogether, these studies support the conclusion that, at least in mice, Glu osteocalcin is the active form of this hormone *in vivo* and that γ-carboxylation negatively regulates both the bioactivity and the bioavailability of osteocalcin. In addition, this work demonstrates a novel role for vitamin K-dependent γ-carboxylation in the control of glucose metabolism and suggests that VKORC1L1 does not act as a VK reductase *in vivo*. Future studies will be oriented toward understanding the physiological function of VKORC1L1 in bone and in other tissues.
